# Distinct Taxonomic and Functional Profiles of the Microbiome Associated With Different Soil Horizons of a Moist Tussock Tundra in Alaska

**DOI:** 10.3389/fmicb.2019.01442

**Published:** 2019-06-27

**Authors:** Binu M. Tripathi, Hye Min Kim1, Ji Young Jung, Sungjin Nam, Hyeon Tae Ju, Mincheol Kim, Yoo Kyung Lee

**Affiliations:** ^1^Korea Polar Research Institute, Incheon, South Korea; ^2^Environmental Safety Research Institute, NeoEnBiz, Bucheon, South Korea

**Keywords:** Arctic tundra, metagenomics, microbiome, permafrost soil, phylogenetic null modeling

## Abstract

Permafrost-underlain tundra soils in Northern Hemisphere are one of the largest reservoirs of terrestrial carbon, which are highly sensitive to microbial decomposition due to climate warming. However, knowledge about the taxonomy and functions of microbiome residing in different horizons of permafrost-underlain tundra soils is still limited. Here we compared the taxonomic and functional composition of microbiome between different horizons of soil cores from a moist tussock tundra ecosystem in Council, Alaska, using 16S rRNA gene and shotgun metagenomic sequencing. The composition, diversity, and functions of microbiome varied significantly between soil horizons, with top soil horizon harboring more diverse communities than sub-soil horizons. The vertical gradient in soil physico-chemical parameters were strongly associated with composition of microbial communities across permafrost soil horizons; however, a large fraction of the variation in microbial communities remained unexplained. The genes associated with carbon mineralization were more abundant in top soil horizon, while genes involved in acetogenesis, fermentation, methane metabolism (methanogenesis and methanotrophy), and N cycling were dominant in sub-soil horizons. The results of phylogenetic null modeling analysis showed that stochastic processes strongly influenced the composition of the microbiome in different soil horizons, except the bacterial community composition in top soil horizon, which was largely governed by homogeneous selection. Our study expands the knowledge on the structure and functional potential of microbiome associated with different horizons of permafrost soil, which could be useful in understanding the effects of environmental change on microbial responses in tundra ecosystems.

## Introduction

About a quarter of the Northern Hemisphere terrestrial ecosystems are covered by permafrost-underlain soils (Zhang et al., 2008), which are key components in the global carbon cycle (McGuire et al., 2009), and stored approximately 50% (~1,700 Pg) of the global below-ground soil organic carbon (Tarnocai et al., 2009). However, this frozen carbon pool is being mobilized due to increased permafrost thaw and deepening of active layer thickness as a result of climate warming (Jorgenson et al., 2001; Osterkamp, 2007; Romanovsky et al., 2010). Though the fate of the stored organic carbon in this region is still unclear, it is expected that the thawing of permafrost soils may trigger an increase in microbial activity promoting decomposition of formerly preserved organic matter and emission of greenhouse gases (DeConto et al., 2012). As soil microbiome plays a crucial role in decomposition and mineralization of organic matter in terrestrial ecosystems, it is important to have a better understanding of permafrost soil microbial ecology in order to improve our prediction of the potential consequences of climate warming on permafrost ecosystem function.

Permafrost soils harbor a diverse microbiome (Jansson and Taş, 2014; Kwon et al., 2019), regardless of subfreezing temperatures and low nutrient availability. In recent years, there has been increasing interest in understanding the diversity and functional potential of microbiome residing in permafrost soils (Yergeau et al., 2010; Mackelprang et al., 2011; Gittel et al., 2014a; Deng et al., 2015; Woodcroft et al., 2018; Tripathi et al., 2018a). Overall, these studies suggest that the variations in composition of permafrost soil microbiomes are related to the corresponding environmental conditions (Gittel et al., 2014a; Deng et al., 2015; Tripathi et al., 2018a). In addition, it has also been recognized that permafrost soil microbiomes have a high potential for nutrient metabolism (Yergeau et al., 2010; Mackelprang et al., 2011; Woodcroft et al., 2018). However, the taxonomy and functional potential of microbiomes associated with different horizons of permafrost soils are still relatively poorly understood.

The assembly of microorganisms in a local community is determined by the interaction of two types of ecological processes: deterministic (abiotic and biotic filtering) and stochastic (e.g., drift and dispersal) (Chase and Myers, 2011; Stegen et al., 2012; Dini-Andreote et al., 2015). Recently, a phylogenetic null modeling approach has been proposed to estimate the relative importance of ecological processes on microbial community assembly (Stegen et al., 2012, 2013; Dini-Andreote et al., 2015). Since then, the relative importance of these ecological processes in shaping the assembly of microbial communities has been studied across a range of ecosystems (Wang et al., 2013; Dini-Andreote et al., 2015; Veach et al., 2016; Graham et al., 2017; Tripathi et al., 2018b); however, permafrost soils have received very limited attention (Tripathi et al., 2018a). This important knowledge gap in permafrost microbial ecology should be addressed by understanding how microbial communities are shaped by the ecological processes in different horizons of permafrost soils.

In this study, we investigated the taxonomy and potential functions of microbiomes, and ecological processes that shape their community composition in different horizons of permafrost soil cores from a moist tussock tundra ecosystem in Council, Alaska. We aimed to address the following questions: (1) how does the taxonomic and functional diversity of microbiomes vary in different horizons of permafrost soil? (2) What are the key factors that influence the composition of microbiomes across different horizons of permafrost soil? (3) How does the relative importance of ecological processes (deterministic vs. stochastic) shape the compositional structure of microbiomes in different horizons of permafrost soil?

## Materials and Methods

### Site Description and Soil Core Sampling

This study was conducted in Council, which is located on the Seward Peninsula in northwestern Alaska (64°51′N, 163°39′W), and field sampling was carried out in July 2014. The mean annual air temperature and precipitation are −3.1 ± 1.4°C and 258 mm, respectively. The sampling site is in discontinuous permafrost region and covered with moist acidic tussock tundra vegetation. The site was dominated with lichen, moss (*Sphagnum* spp.), bog blueberry (*Vaccinium uliginosum*), and water sedge (*Carex aquatilis*) (Park and Lee, 2014). The soil was classified as Histic-turbic Cryosols with a WRB system (Typic Histoturbels with a US soil Taxonomy).

**Figure 1:**
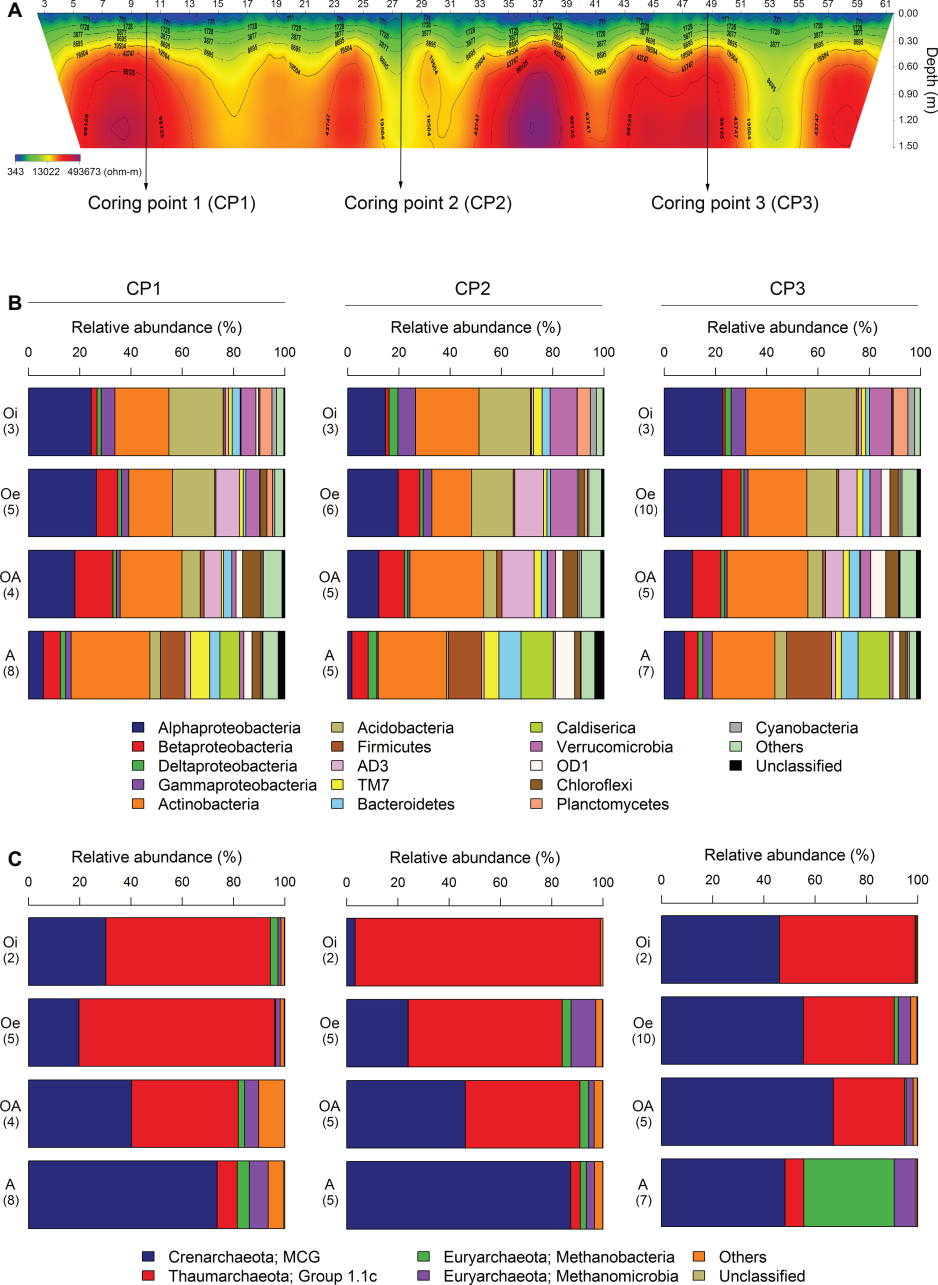
**(A)** The 2D-electrical resistivity tomographic (ERT) survey result of three coring points. Average relative abundance of dominant **(B)** bacterial and **(C)** archaeal taxa associated with different horizons of soil cores sampled from three coring points. Number of samples analyzed in each horizon is given in brackets.

A 2D-electrical resistivity tomographic (ERT) survey was performed using ABEM Terrameter LS system capable of automatic measurement of up to 64 points in order to examine the spatial distribution of permafrost. Based on the ERT result, we choose three points for collecting soil cores (Figure 1A). Coring points (CP) 1 and 3 have lower soil water contents or an ice crystal state (electric resistivity: ~100 kΩ) compared to CP2 (electric resistivity: ~10 kΩ) (Figure 1A). The thaw depth at the time of sampling was between 30 and 40 cm. From each site, three soil cores (up to 1.5 m) were collected using a SIPRE coring auger (7.6 cm in diameter, John’s Machine Shop, Fairbanks, AK). All the sampled cores were placed in ice coolers and transported to the laboratory freezer (−20°C) at the University of Alaska, Northwest Campus Nome, Alaska. Soil cores were shipped in frozen state to the laboratory at Korea Polar Research Institute, Korea by using an ice-breaking research vessel ARAON, where these were stored at −20°C until further processing.

### Soil Analyses

Soil horizons of each soil core were divided into Oi and Oe horizons (organic materials), and OA and A horizons. The OA horizon was placed in between organic and mineral A layers, and most A horizons were found below 50 cm depth. We defined here Oi horizon as “top soil,” and Oe, OA, and A horizons as “sub-soil.” Soil texture was determined by the pipette method (Gee and Bauder, 1986). Electrical conductivity (EC) and soil pH were measured in a soil-water suspension (1:5 ratio, w/v) using a pH/EC meter (Orion Star A215, Thermo Scientific, Waltham, MA, USA). Water content (WC) was determined by measuring the weight change in soils after oven-drying at 105°C for 48 h. Total carbon (TC) and total nitrogen (TN) contents in soils were measured using an elemental analyzer (FlashEA 1,112 Thermo Electron corporation, Waltham, Massachusetts, USA). For NH_4_^+^-N and NO_3_^−^-N contents analysis, fresh soil was extracted using 2 M KCl solution and subsequently filtrates were analyzed on an auto-analyzer (QuAAtro; Seal Analytical, Norderstedt, Germany). Fresh soil was mixed with deionized water and then filtered through Whatman filter paper #42 firstly and then 0.45-μm filter to acquire water extractable carbon (WEC) and nitrogen (WEN).

### DNA Extraction, 16S rRNA Gene Amplification, and Sequencing

Soil DNA was extracted from each sample with 0.50 g of soil using FastDNA™ SPIN Kit (MP Biomedicals, Santa Ana, CA, USA) in triplicates to obtain sufficient DNA quantity. The V1-V3 regions of bacterial 16S rRNA gene were amplified using primer pair V1-9F (5′-GAGTTTGATCMTGGCTCAG-3′) and V3-541R (5′-WTTACCGCGGCTGCTGG-3′) (Chun et al., 2010). Archaeal 16S rRNA gene was amplified by targeting V3-V6 regions with primer pair S-D-Arch-0519-a-S-15 (5′-CAGCMGCCGCGGTAA-3′) and S-D-Arch-1,041-a-A-18 (5′-GGCCATGCACCWCCTCTC-3′) (Klindworth et al., 2013). DNA sequencing was performed at Macrogen Incorporation (Seoul, South Korea) using a 454 GS FLX Titanium pyrosequencing system (Roche).

### Sequence Processing

The resulting 16S rRNA gene sequences were processed in Mothur v.1.39.5 (Schloss et al., 2009). At first, sequences with average quality score < 25, with read length < 200 bp, with homopolymers longer than 8 nt, and with base-call and barcode ambiguity were removed. Next, the quality filtered unique sequences were aligned against a SILVA alignment^1^, and subsequently the sequence alignments were pre-clustered within a distance of 2 bp using a pseudo-single linkage algorithm (Huse et al., 2010). Chimeric 16S rRNA gene sequences were identified and removed *via* the Chimera Uchime algorithm in *de novo* mode (Edgar et al., 2011). Taxonomic classifications of bacterial and archaeal 16S rRNA gene sequences were performed using the naive Bayesian classifier (80% bootstrap cutoff with 1,000 iterations) against an EzTaxon-extended database (Kim et al., 2012). Sequences were clustered into operational taxonomic units (OTUs) at a threshold of ≥97% sequence similarity using the average neighbor clustering algorithm (Schloss and Westcott, 2011). To avoid spurious results due to pyrosequencing errors, the entire singleton OTUs were removed prior to subsequent analyses. To account for differences in sequencing depth, 1,073 and 116 sequences were randomly sampled from each sample of bacterial and archaeal sequence datasets, respectively. All the sequences were deposited in Sequence Read Archive (SRA) at NCBI under the BioProject ID PRJNA 513409.

### Phylogenetic Analyses

The representative 16S rRNA gene sequences of bacterial and archaeal OTUs were used to construct a maximum-likelihood tree using FastTree program (Price et al., 2010). To analyze the phylogenetic assembly within each community, we calculated standardized effect size measure of mean nearest taxon distance (SES.MNTD) in Picante R package using the null model “taxa.labels” with 999 randomization (Kembel et al., 2010). Significant negative values of SES.MNTD indicate that co-occurring OTUs are phylogenetically more closely related than expected under a random model of community assembly (clustering), whereas significant positive values indicate co-occurring OTUs are phylogenetically more distantly related than expected (over dispersion) (Webb et al., 2002).

Phylogenetic β-diversity was calculated using mean nearest taxon distance (βMNTD) in Picante R package, which measures the degree of phylogenetic similarity of closely related OTUs in two communities. Furthermore, to infer the relative influence of ecological processes that govern the assembly of microbial communities, a previously developed null modeling approach was used to calculate β-nearest taxon index (βNTI) (Stegen et al., 2012, 2013; Dini-Andreote et al., 2015). βNTI is the difference between observed βMNTD and the mean of the null distribution of βMNTD measured in units of its standard deviation. The pairwise comparisons of βNTI < −2 or > +2 indicate significantly less than (homogeneous selection) or greater than expected phylogenetic turnover (variable selection) (Dini-Andreote et al., 2015). We further calculated the Bray-Curtis-based Raup-Crick metric (RC_bray_) as described by Stegen et al. (2013, 2015) to quantify the relative contribution of various stochastic processes, that is, homogenizing dispersal, dispersal limitation, and drift on pairwise comparisons with |βNTI| < 2. The fraction of pairwise comparisons with |βNTI| < 2 and RC_bray_ < −0.95 indicate less than expected turnover due to homogenizing dispersal, whereas the fraction of pairwise comparisons with |βNTI| < 2 and RC_bray_ > +0.95 indicate more than expected turnover due to dispersal limitation. The fraction of pairwise comparisons with |βNTI| < 2 and |RC_bray_| < 0.95 indicate that compositional turnover is not determined by any single process (referred as “undominated” processes in Stegen et al. (2015)).

### Shotgun Metagenome Sequencing and Data Analyses

We pooled extracted DNA of replicate samples of each of the four horizons of each core to collect sufficient amount of DNA for metagenome sequencing using Illumina HiSeq2000 platform (2 × 150 bp) (Illumina, Inc.) at Macrogen Incorporation (Seoul, South Korea). For quality filtering, the resulting paired-end sequences were uploaded to Metagenomics Rapid Annotation (MG-RAST) server (Meyer et al., 2008). In MG-RAST server, the paired-end sequences were joined with “retain” option, which allows retention of non-overlapping paired reads. After that, the sequencing adapters were trimmed and sequences were quality filtered and denoised.

The MG-RAST quality control-passed reads were aligned against latest NCBI-nr database (downloaded in May, 2018) using the BLASTX algorithm in DIAMOND v.0.9.10.111 (Buchfink et al., 2014), with default parameters. For functional profiling, Kyoto Encyclopedia of Genes and Genomes (KEGG) mapping files were used to map aligned reads to the KEGG functions in MEtaGenome ANalyzer (MEGAN) v.6.5.7 (Huson et al., 2007). Taxonomic classification of metagenomic reads annotated to methyl coenzyme M reductase (*mcrA*), particulate methane monooxygenase (*pmoA*), and soluble methane monooxygenase (*mmoX*) genes was performed in GraftM v.0.11.1 using *mcrA*-, *pmoA*-, and *mmoX*-specific GraftM packages (gpkg) (Boyd et al., 2018). The GraftM assigns taxonomy to amino acid sequences by placing them into the reference phylogenetic trees built beforehand for specific protein family (e.g., McrA). All the shotgun metagenomic sequences are deposited in the MG-RAST server under project ID mgp21063^2^.

### Statistical Analyses

Based on the abundance profile, the functional genes (KEGG level 3) with significantly differential abundances across soil horizons were determined using DESeq2 R package (Love et al., 2014). Gene abundances are considered significantly different between soil horizons if the false discovery rate (FDR)-adjusted *p* value is less than 0.05. To test the variations in soil physico-chemical parameters, alpha-diversity indices, taxonomic and functional distributions of microbiomes between soil horizons and coring points, we used analysis of variance (ANOVA) followed by additional *post hoc* Tukey’s tests where required. To detect the effect of soil horizon profile on physico-chemical parameters, a principal component analysis (PCA) was performed in Canoco 5.0 (Biometrics, Wageningen, The Netherlands), followed by a permutational multivariate ANOVA (PERMANOVA) test. We used non-metric multi-dimensional scaling (NMDS) plots to visualize the pairwise Bray-Curtis dissimilarities between communities. A PERMANOVA test was carried out to compare the Bray-Curtis dissimilarities between soil horizons and coring points. Additional *post hoc* tests were performed after PERMANOVA analyses when significant differences were observed (*p* < 0.05). PERMANOVA analyses were performed in PRIMER 6.0 with the PERMANOVA+ add-on (Clarke and Gorley, 2006; Anderson et al., 2008). We performed canonical correspondence analysis (CCA) to test which soil properties best explained the variations in composition of bacterial and archaeal OTUs using Canoco 5.0 (Ter Braak and Šmilauer, 2012), applying forward selection and the Monte Carlo permutation test with 999 random permutations. In addition, we used “procrustes” function in vegan R package (Oksanen et al., 2007) to assess congruence between ordinations of microbial community structure and soil physico-chemical parameters and the significance of the Procrustes statistic was tested by 999 permutations with “protest” function. Furthermore, we used Spearman rank correlations to examine the influence of soil properties on each dominant OTUs of bacteria and archaea. The OTUs with relative abundances greater than 0.5% were identified as dominant OTUs.

## Results

### Soil Characteristics

All the measured soil physico-chemical parameters varied significantly between soil horizons (Supplementary Table S1). Electrical conductivity, WC, TC, TN, carbon-to-nitrogen ratio (C:N), NO_3_^−^-N, WEC and WEN content were highest in top soil horizon (Oi) (Supplementary Table S1), whereas pH and NH_4_^+^-N, WEC-to-TC (WEC:TC) and WEN-to-TN (WEN:TN) ratios were highest in sub-soil horizons (Supplementary Table S1). Principal component analysis on soil physico-chemical parameters placed top soil and sub-soil horizons at different positions on ordination (Supplementary Figure S1). PERMANOVA analysis showed soil physico-chemical properties varied significantly between soil horizons (*p* < 0.05; Supplementary Table S2); however, the effect of coring point was not significant on soil physico-chemical properties (*p* = 0.26; Supplementary Table S2).

### Community Structure and Diversity

The most dominant bacterial phylum detected across all soil samples was *Actinobacteria*, accounting for ∼24% of all sequences, followed by *Proteobacteria* (Alpha-, Beta-, Gamma-, and Delta-classes), *Acidobacteria, Firmicutes*, AD3, TM7, *Bacteroidetes, Caldiserica, Verrucomicrobia*, OD1, *Chloroflexi, Planctomycetes*, and *Cyanobacteria* (Figure 1B). The relative abundance of these dominant bacterial phyla showed similar horizon-associated pattern across all cores (Figure 1B). Furthermore, the relative abundance of most of the dominant bacterial phyla varied significantly (*p* < 0.05) between soil horizons (Table 1), with *Proteobacteria* (Alpha- and Gamma-classes), *Acidobacteria, Verrucomicrobia, Planctomycetes*, and *Cyanobacteria* being more abundant in top soil horizon, whereas sub-soil horizons were dominated by *Betaproteobacteria, Firmicutes*, AD3, *Bacteroidetes, Caldiserica*, OD1, and *Chloroflexi*. The relative abundance of dominant archaeal taxa (at class level) displayed similar horizon-associated pattern in core 1 and core 2, but showed a different pattern in core 3 (Figure 1C). The archaeal communities across all soil samples were dominated by the members of Miscellaneous Crenarchaeotal Group (MCG) (Figure 1C). In addition, *Thaumarchaeota* Group 1.1c, *Methanobacteria*, and *Methanomicrobia* were also present across all soil samples (Figure 1C). Out of these, the relative abundance of *Thaumarchaeota* Group 1.1c was significantly (*p* < 0.05) higher in top soil horizon, whereas the rest of the archaeal taxa (MCG, *Methanobacteria* and *Methanomicrobia*) were dominated in sub-soil horizons (Table 1).

**Table 1:** Relative abundance of dominant bacterial and archaeal taxa in different horizons across all soil cores.

	Oi	Oe	OA	A
**Bacterial taxa**				
*Alphaproteobacteria*	20.7 ± 6.5 (ab)	22.6 ± 11.0 (a)	12.6 ± 6.6 (bc)	5.6 ± 4.9 (c)
*Betaproteobacteria*	1.5 ± 1.1 (c)	7.9 ± 4.5 (ab)	11.2 ± 5.1 (a)	6.3 ± 4.6 (b)
*Gammaproteobacteria*	6.0 ± 1.9 (a)	2.4 ± 1.7 (b)	1.0 ± 0.5 (b)	2.4 ± 2.2 (b)
*Actinobacteria*	23.0 ± 5.8	19.5 ± 8.7	28.9 ± 14.8	27.1 ± 10.1
*Acidobacteria*	20.4 ± 6.8 (a)	14.1 ± 7.5 (b)	5.7 ± 2.9 (c)	3.5 ± 3.7 (c)
*Firmicutes*	0.7 ± 0.7 (b)	0.7 ± 0.5 (b)	2.2 ± 2.7 (b)	13.4 ± 9.0 (a)
AD3	1.1 ± 0.6 (b)	8.8 ± 5.8 (a)	8.4 ± 8.3 (a)	1.6 ± 1.5 (b)
TM7	2.1 ± 1.2	1.8 ± 0.9	2.0 ± 1.6	5.4 ± 10.9
*Bacteroidetes*	2.6 ± 1.2 (b)	1.9 ± 1.8 (b)	3.5 ± 2.4 (ab)	5.9 ± 3.8 (a)
*Caldiserica*	0.1 ± 0.2 (b)	0.1 ± 0.2 (b)	1.3 ± 4.6 (b)	10.1 ± 8.0 (a)
*Verrucomicrobia*	8.3 ± 4.5 (a)	6.3 ± 4.4 (a)	2.9 ± 1.7 (b)	1.4 ± 1.2 (b)
OD1	0.4 ± 0.9 (b)	1.9 ± 2.5 (ab)	3.7 ± 2.9 (ab)	4.2 ± 4.6 (a)
*Chloroflexi*	0.3 ± 0.6 (c)	2.7 ± 1.9 (b)	5.6 ± 2.4 (a)	2.8 ± 1.5 (b)
*Planctomycetes*	5.2 ± 1.2 (a)	1.2 ± 1.0 (b)	0.4 ± 0.3 (c)	0.4 ± 0.4 (c)
*Cyanobacteria*	2.3 ± 0.6 (a)	0.7 ± 0.3 (b)	0.5 ± 0.5 (b)	0.4 ± 0.5 (b)
**Archaeal taxa**				
MCG	26.5 ± 30.1 (b)	38.6 ± 25.0 (b)	52.8 ± 31.9 (ab)	68.4 ± 24.7 (a)
*Thaumarchaeota* Group 1.1c	70.9 ± 32.5 (a)	51.9 ± 27.1 (ab)	36.2 ± 32.0 (b)	6.3 ± 9.2 (c)
*Methanobacteria*	1.1 ± 2.4 (b)	1.7 ± 2.8 (b)	2.0 ± 1.9 (ab)	15.6 ± 27.4 (a)
*Methanomicrobia*	0.4 ± 1.0 (b)	5.3 ± 8.9 (a)	4.5 ± 5.5 (a)	6.0 ± 8.6 (a)

Data represent mean ± standard deviation. Within each row, values followed by different letters (in brackets) are statistically different (*p* < 0.05) according to one-way ANOVA and Tukey’s HSD test.

**Figure 2:**
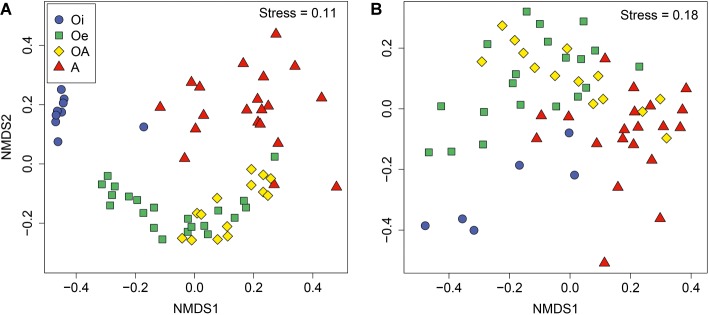
Non-metric multidimensional scaling plots based on Bray–Curtis dissimilarities of **(A)** bacterial and **(B)** archaeal communities between samples of different soil horizons.

**Figure 3:**
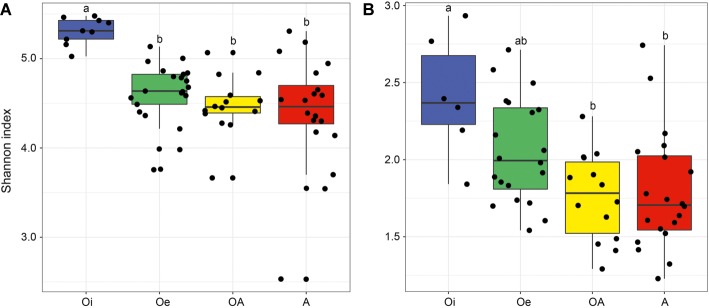
Box plot showing variation in Shannon diversity index of **(A)** bacterial and **(B)** archaeal communities according to soil horizons.

NMDS ordinations based on Bray–Curtis dissimilarity matrix showed that both bacterial and archaeal communities were clustered based on soil horizon (Figure 2), and PERMANOVA analyses revealed that the community composition of bacteria and archaea was significantly different in each horizon (*p <* 0.05; Supplementary Table S2), except archaeal community composition in Oe and OA soil horizons (*p =* 0.10; Supplementary Table S2). However, coring point did not significantly influence the microbial community composition (*p* > 0.05; Supplementary Table S2). The alpha-diversity (Shannon index) of bacterial and archaeal 16S rRNA gene OTUs was significantly (*p* < 0.05) higher in top soil horizon compared to sub-soil horizons (Figure 3).

**Figure 4:**
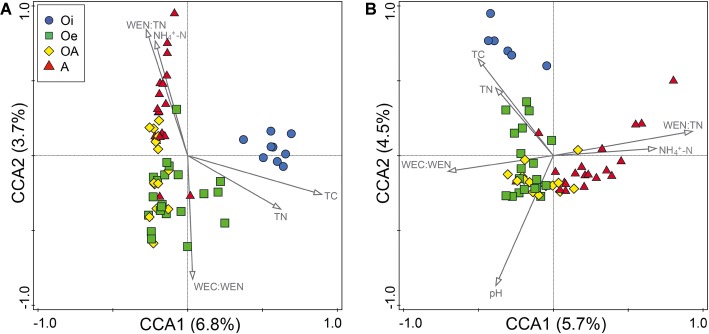
Canonical correspondence analysis of **(A)** bacterial and **(B)** archaeal OTU compositions in samples of four different soil horizons.

In CCA, we found that of all the measured environmental variables, TC, TN, WEC:WEN, WEN:TN, and NH_4_^+^-N appeared to best explain the variations in community composition of both bacteria and archaea (Figure 4). Whereas, soil pH was significantly related with only archaeal community composition (Figure 4). However, taken together these soil properties only explained 16 and 19% of the total variations in bacterial and archaeal community composition, respectively. The Procrustes analysis comparing spatial fit between ordinations of microbial community structure (NMDS plots) and soil physico-chemical parameters (PCA plots) showed concordance (*p* < 0.05; Supplementary Figure S2), indicating that across all cores bacterial and archaeal communities were strongly associated with soil physico-chemical properties. We further explored the correlation between environmental variables and dominant microbial OTUs and found that most of the dominant OTUs were significantly associated with all measured environmental variables (Supplementary Figure S3). Based on these associations, the environmental variables were mainly clustered into three groups and displayed different correlation patterns (Supplementary Figure S3). For example, bacterial OTUs belonging to the order *Acidobacteriales, Rhizobiales, Acidimicrobiales*, and *Solirubrobacterales* were positively correlated with C:N ratio, TC, TN, EC, NO_3_^−^-N, WEN, WC, and WEN (group I), and negatively correlated with depth (group II) and pH (group III). Additionally, OTUs belonging to *Clostridiales, Rubrobacterales, Bacteroidales*, and *Caldisericales* were positively correlated with depth, NH_4_^+^-N, WEC:TC, and WEN:TN ratio (group II) and negatively correlated with C:N ratio, TC, TN (group I), and WEC:WEN ratio (group III). The archaeal OTUs also showed similar patterns with environmental variables (Supplementary Figure S3), for instance, OTUs belonging to *Methanobacterium* were positively correlated with Depth, EC, WEN, NH_4_^+^-N, WEC:TC, and WEN:TN ratio (group I) and negatively correlated with pH (group III). In addition, OTUs belonging to *Thaumarchaeota* Group 1.1c were positively correlated with TC, TN, C:N, ratio, NO_3_^−^-N, WC, and WEC (group II) and negatively correlated with depth (group I) and pH (group III).

### Potential Functional Genes

**Figure 5:**
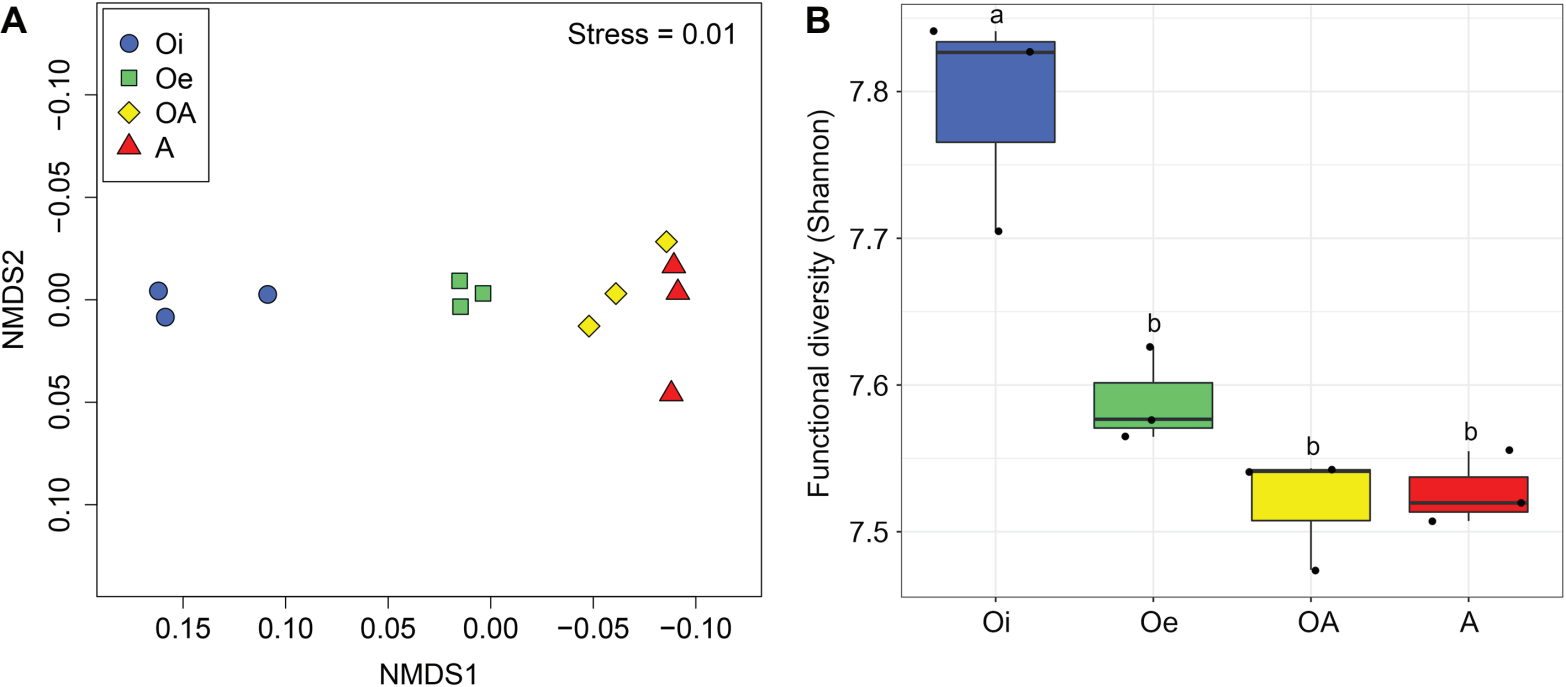
**(A)** Composition and **(B)** diversity of functional genes (KEGG level 3) in different soil horizons of permafrost soil cores.

**Figure 6:**
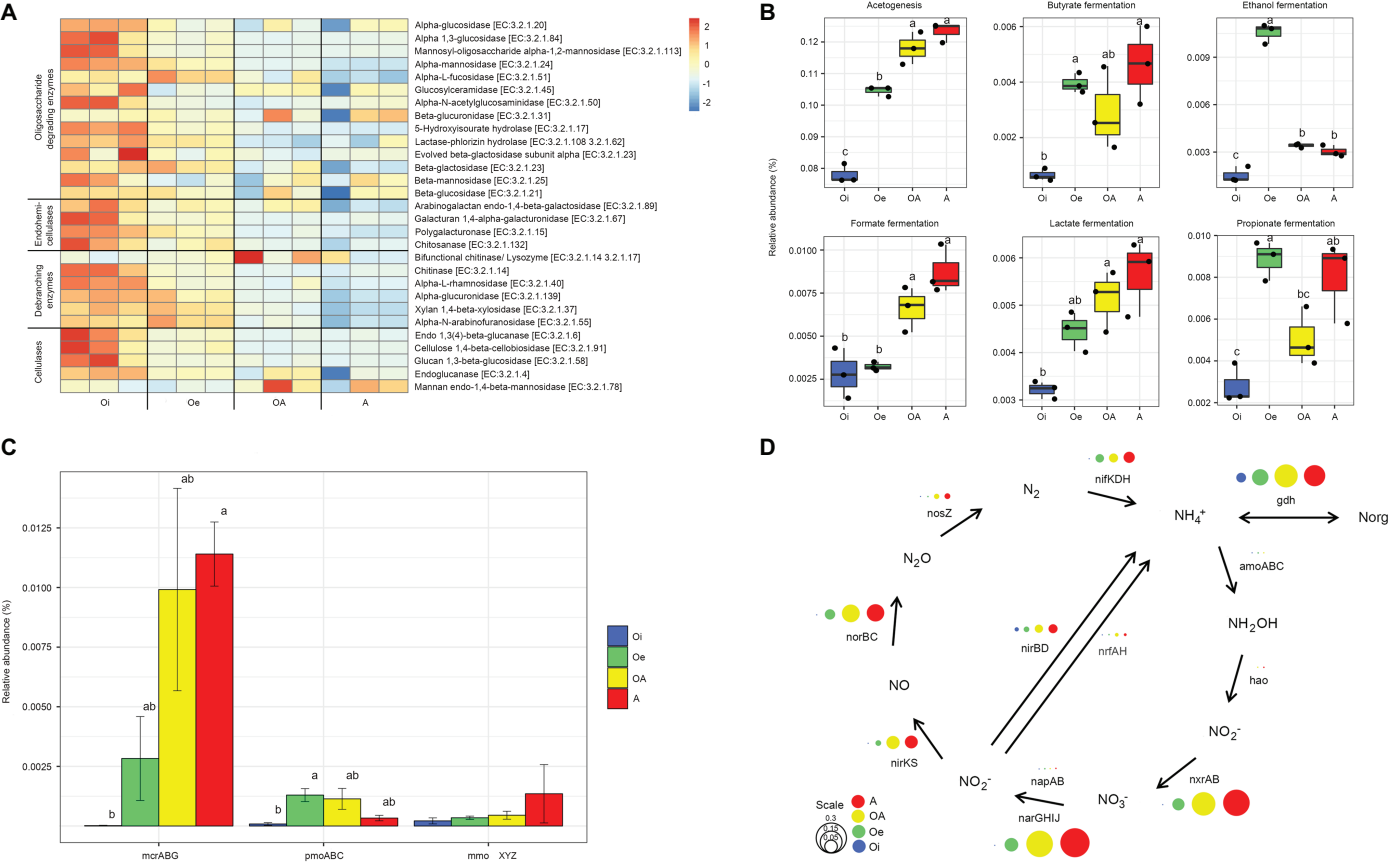
Genetic potential for carbon and N metabolism in different horizons of permafrost soil cores. **(A)** Heatmap of scaled relative abundance of genes encoding carbohydrate-active enzymes (CAZymes). **(B)** Relative abundance of genes involved in acetogenesis and fermentation. **(C)** Relative abundance of genes encoding sub-units of key enzymes involved in methanogenesis (methyl coenzyme M reductase, *mcrABG*) and methanotrophy (particulate methane monooxygenase-*pmoABC* and soluble methane monooxygenase-*mmoXYZ*). **(D)** Relative abundance of genes associated with N cycle. The circle size corresponds to the relative abundance of each gene.

The composition of functional genes (KEGG level 3) was structured according to soil horizons (PERMANOVA, *p* < 0.05; Figure 5A). The alpha-diversity (Shannon index) of functional genes varied significantly (*p* < 0.05) between soil horizons, with top soil horizon having significantly higher functional gene diversity than sub-soil horizons (Figure 5B). A total of 14,081 unique KEGG functional genes (level 3) were identified, and out of these, 6,802 genes varied significantly across soil horizons (adjusted *p* < 0.05, DESeq2, Supplementary Data S1). For the sake of simplicity, we focused only on genes involved in carbon and nitrogen metabolism. The relative abundance of functional genes encoding carbohydrate-active enzymes (CAZymes) was higher in top soil horizon compared to sub-soil horizons (Figure 6A). However, the functional genes involved in acetogenesis and fermentation were significantly (*p* < 0.05) more abundant in sub-soil horizons (Figure 6B). When we looked at the functional genes involved in methane metabolism, we found that the relative abundance of genes encoding sub-units of key enzymes involved in methanogenesis (*mcrABG*) and methanotrophy (*pmoABC* and *mmoXYZ*) were more abundant in sub-soil horizons compared to top soil horizon (Figure 6C). Taxonomic classification of *mcrA* reads showed that most of the methanogenic taxa belonged to “*Candidatus* Methanoflorens” (Supplementary Figure S4), whereas upland soil cluster α (USCα) and *Methylococcaceae* were dominant in *pmoA* and *mmoX* reads, respectively (Supplementary Figure S4).

The relative abundance of functional genes involved in nitrogen cycling also varied between soil horizons, with sub-soil horizons harboring more nitrogen cycling genes than top soil horizon (Figure 6D). Sub-soil horizons had higher genetic potential for nitrogen fixation, nitrification, and denitrification. The genes involved in conversion of ammonium to organic nitrogen (*gdh*) were also relatively more abundant in sub-soil horizons than in top soil horizon.

### Community Assembly Processes

**Figure 7:**
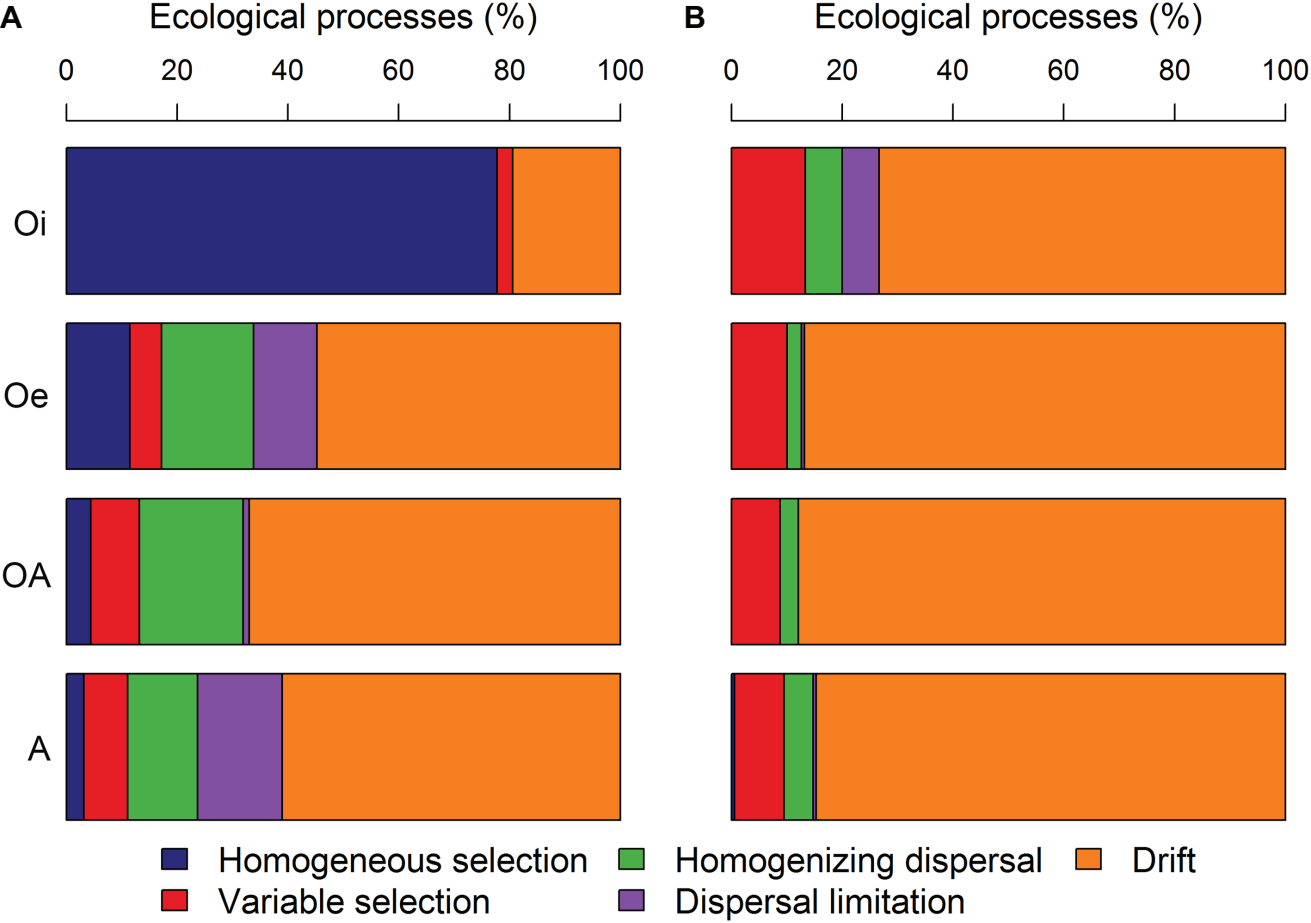
The contribution of various deterministic (homogeneous and variable selection) and stochastic processes (homogenizing dispersal, dispersal limitation and drift) that governed the assembly of **(A)** bacterial and **(B)** archaeal communities.

The comparison of pairwise βNTI and RC_bray_ distances within each soil horizon showed that bacterial community assembly was more deterministic (homogeneous selection) in top soil horizon and shifted toward stochastic assembly with undominted fraction becoming more dominant in sub-soil horizons (Figure 7A). While, archaeal community assembly was primarily structured by stochastic processes in all soil horizons with dominance of undominated fraction (Figure 7B).

## Discussion

Our results showed that the communities of this moist tussock tundra soil microbiomes were significantly different between top soil and sub-soil horizons. This result is in agreement with the findings of previous studies which reported that composition of microbial communities varied significantly between different soil layers along the depth of permafrost-underlain soil cores (Gittel et al., 2014a; Deng et al., 2015; Taş et al., 2018; Tripathi et al., 2018a). The availability of labile carbon and nitrogen is usually highest in top soil horizon as it represents the major location for root exudates, which typically favors the growth of copiotrophic microorganisms (Fierer et al., 2007). In line with this hypothesis, we found that the relative abundance of copiotrophic microorganisms such as Alpha- and Gamma-subdivisions of *Proteobacteria* was highest in top soil horizon. However, the oligotrophic/copiotrophic framework was not able to explain the contrasting distribution patterns of several other taxa, which were designated as either oligotrophic (*Acidobacteria, Verrucomicrobia*, and *Planctomycetes*) or copiotrophic (*Betaproteobacteria* and *Bacteroidetes*) (Fierer et al., 2007, 2012; Ramirez et al., 2012; Leff et al., 2015). It is more likely that the observed distribution patterns are influenced by the physiology of the corresponding microbial lineages and soil environmental conditions. The relative abundance of *Actinobacteria* remained similar in all four soil horizons; the members of the *Actinobacteria* have been reported to dominate in both active and permafrost layer of Arctic tundra soils (Gittel et al., 2014b; Deng et al., 2015). It has been suggested that *Actinobacteria* are successful in colonizing permafrost soils because they have adapted to harsh abiotic conditions and have capabilities to degrade complex organic compounds, such as cellulose and lignin (DeAngelis et al., 2011; Gittel et al., 2014b). For example, particularily the relative abundance of *Rubrobacterales* substantially increased in permafrost layer. Members of *Rubrobacterales* are known to have tolerance to radiation and dessication (Ferreira et al., 1999), and commonly occur in Arctic permafrost soils (Mackelprang et al., 2011; Wilhelm et al., 2011). The dominance of *Acidobacteria* in top soil horizon could be related to pH, which was relatively more acidic in top soil horizon compared to sub-soil horizons (Supplementary Table S1). We also observed strong negative correlation between one of the dominant acidobacterial OTUs and soil pH (Supplementary Figure S3). *Acidobacteria* are known to dominate acidic and organic rich tundra soils (Männistö et al., 2013), where they have been shown to play key role in degradation of large polysaccharides (Woodcroft et al., 2018). Members of the phyla *Verrucomicrobia* and *Planctomycetes* are reported to be abundant in *Sphagnum*-dominated top layers of Arctic peat soils (Tveit et al., 2013), and they have the ability to degrade various plant-derived organic matter (Hernández et al., 2015; Moore et al., 2015). Within *Betaproteobacteria*, the microaerophilic iron-oxidizing genus *Gallionella* was found to dominate the deeper soil horizons, and previous studies have demonstrated an increase in relative abundance of this iron-oxidizing lineage in ferrous iron [Fe(II)]-rich deeper soil horizons (Müller et al., 2018). In agreement with other studies (Gittel et al., 2014a; Deng et al., 2015), we also observed dominance of fermentative members of *Bacteroidetes* (*Bacteroidia*) and *Firmicutes* (*Clostridia*) in deeper soil horizons, which indicates high anaerobic degradation potential of deeper soil horizons. The unknown lineages of *Chloroflexi* (GQ396871_g) and *Caldiserica* (EU266853_g) were dominant in deeper soil horizons (data not shown). These poorly characterized bacterial phyla together with other candidate phyla such as AD3, TM7, and OD1 have been shown to dominate deeper horizons of permafrost soils (Jansson and Taş, 2014; Taş et al., 2014; Monteux et al., 2018; Tripathi et al., 2018a). The functions of these poorly described bacterial phyla should be further investigated through culture-dependent and culture-independent approaches to better understand ecological processes in the deeper permafrost soil horizons.

The archaeal community was dominated by *Thaumarchaeota* Group 1.1c in top soil horizon and their relative abundance gradually decreased in sub-soil horizons. This may be the result of the relatively lower pH of top soil horizon than that of sub-soil horizons. The members of *Thaumarchaeota* Group 1.1c are commonly reported from acidic soils (Kemnitz et al., 2007; Nicol et al., 2007), and their relative abundance sharply declines with increase in pH (Lehtovirta et al., 2009; Tripathi et al., 2013). Strong negative correlations were also observed in this study between some of the dominant *Thaumarchaeota* Group 1.1c OTUs and soil pH (Supplementary Figure S3). The deeper soil horizons were dominated by the representatives of MCG (*Bathyarchaeota*), which are anaerobic heterotrophs and known to assimilate a wide variety of organic carbon compounds (Lazar et al., 2016; Xiang et al., 2017). The deeper soil horizons also harbored members of methanogenic genera (*Methanobacterium*, “*Ca.* Methanoflorens,” and *Methanosarcina*). *Methanobacterium* and “*Ca.* Methanoflorens” are hydrogenotrophic methanogens that utilize hydrogen and carbon dioxide or formate as substrates for methanogenesis (Mondav et al., 2014; Oren, 2014a), and these methanogens are known to form syntrophic associations with several bacterial species to facilitate degradation of organic matter in anoxic environments (Woodcroft et al., 2018). Whereas, *Methanosarcina* is metabolically more flexible, capable of all three pathways of methanogenesis, that is, hydrogenotrophic, acetoclastic and methylotrophic (Oren, 2014b). All these methanogens have been shown to dominate the deeper soil layers of Arctic tundra (Tripathi et al., 2018a). The dominance of methanogenic taxa in deeper soil horizons indicates that these soils have higher potential for methane emission.

Bacterial and archaeal diversity was significantly lower in sub-soil horizons compared to top soil horizon, which could be related with harsh abiotic environmental conditions (e.g., low to subzero temperatures, high water content, and anoxia due to water logging) of sub-soil horizons, that impose a strong ecological filter on colonization of microbiota leading to reduce the diversity of microbial communities.

The different horizons of the permafrost-underlain Arctic tundra soil represent stratifically heterogeneous environment due to vertical gradient in soil physico-chemical parameters and seasonal freeze-thaw cycles in the active soil layer. We found that the composition of bacterial and archaeal communities across soil horizons was associated with depth-related variations in soil physico-chemical properties (Supplementary Figure S2). Similar vertical distribution patterns were observed in previous studies on permafrost soil microbial communities (Deng et al., 2015; Tripathi et al., 2018a). However, a large fraction of the variations in bacterial (84%) and archaeal (81%) communities remained unexplained, suggesting that other factors such as stochasticity and unmeasured environmental variables could impact the variations in community composition.

The majority of organic matter stored in the surface soils is derived from plant polymers such as cellulose and hemicellulose. The degradation of these high-molecular weight polysaccharides to oligomeric and monomeric sugars is a key step in microbial decomposition of soil organic matter (Kotsyurbenko, 2005). The top soil horizon had higher genomic potential for degradation of both polysaccharides and oligosaccharides (Figure 6A). The organic matter inputs from plants and favorable environmental conditions for microbial growth in top soil horizon might have contributed to the observed increase in the relative abundance of genes involved in organic matter decomposition. It has been reported earlier that top horizon of Arctic soils has higher enzymatic activities of carbohydrate metabolism compared to sub-soil horizons (Gittel et al., 2014a). Furthermore, our metagenome data showed a significant increase in relative abundance of genes involved in fermentation and acetogenesis in sub-soil horizons (Figure 6B). Fermentation and acetogenesis pathways are key to the degradation of monosaccharides in sub-soil horizons and produce low-molecular weight alcohols and organic acids together with hydrogen and carbon dioxide (Conrad, 1999; Ye et al., 2012). We also found that the genes involved in methanogenesis (*mcrABG*) and methanotrophy (*pmoABC* and *mmoXYZ*) were more abundant in sub-soil horizons (Figure 6C). Methanogenesis is the terminal step in anaerobic decomposition of organic carbon and performed by archaeal methanogens, whereas methanotrophy is mostly carried out by bacterial methanotrophs which act as a sink for methane before it gets released to the atmosphere (Le Mer and Roger, 2001). As methanogenesis is an obligately anaerobic process, the higher relative abundance of *mcrABG* genes in sub-soil horizons could be related with its low oxygen and redox levels. The taxonomy of *mcrA* gene showed that “*Ca.* Methanoflorens,” a hydrogenotrophic methanogen, was dominant in these soils, which has been identified as major contributor to methane production in Arctic tundra soils (Mondav et al., 2014; Woodcroft et al., 2018). The *pmoA* reads were dominated by USCα (Knief, 2015), which oxidizes atmospheric methane aerobically and has recently been identified in a wide range of terrestrial ecosystems globally (Tveit et al., 2019), including permafrost soils (Lau et al., 2015; Singleton et al., 2018). *Methylococcaceae* was predominant in *mmoX* gene taxonomy, which is known to oxidize methane in microaerophilic conditions (Kits et al., 2015), and can remain active in deeper permafrost layers (Tveit et al., 2014; Singleton et al., 2018). The dominance of methanotrophic taxa adapted to function in both aerobic and microaerophilic conditions in sub-soil horizons suggests that a substantial portion of methane might get oxidized by methanotrophs before being emitted into the atmosphere.

Nitrogen availability regulates the microbial decomposition of organic matter and subsequent release of greenhouse gases; however, the Arctic terrestrial environments are generally considered nitrogen limited (Shaver and Chapin, 1980). Therefore, the microorganisms involved in the process of nitrogen cycling (nitrogen fixation, nitrification, denitrification, and nitrogen assimilation) in permafrost soils might play a pivotal role in the microbial response to global warming. Similar to other studies (Keuper et al., 2012; Salmon et al., 2018), we found higher amounts of available nitrogen as a form of ammonium and WEN in deeper sub-soil horizons despite the lower content of TN (Supplementary Table S1). Our results also showed that the sub-soil horizons had higher genetic potential for nitrogen cycling (Figure 6D), which indicates that microorganisms harboring these genes are already present in permafrost soils and could increase nitrogen cycling rates in these soils provided with favorable environmental conditions. The nitrogen assimilation genes, especially those involved in the conversion of ammonium to organic nitrogen (*gdh*), were enriched in sub-soil horizons, suggesting that microorganisms residing in this nitrogen-poor environment are poised to take up nitrogen as it becomes available. Our results also showed that the genetic potential for denitrification was enriched in sub-soil horizons (Figure 6D). Higher genetic potential for denitrification was also detected in other metagenomic studies on permafrost soils (Lipson et al., 2013).

Understanding the ecological processes that shape the assembly of permafrost soil microbiome is critical in order to fill an important knowledge gap in permafrost microbial ecology and predict the ecosystem responses to thaw. Our null modeling analyses revealed a more deterministic bacterial community assembly in top soil horizon with a strong influence of homogeneous selection (Figure 7A). This might have resulted due to low variation in soil physico-chemical properties within samples of top soil horizon (Supplementary Figure S1), as a consistent selective environment results in similar community composition at local scales through homogeneous selection (Dini-Andreote et al., 2015). However, in sub-soil horizons, bacterial community assembly was largely driven by stochastic processes (Figure 7A), which indicates that the strength of environmental selection may have weakened in sub-soil horizons. It has been reported that microbial movement is restricted within permafrost soil layer (Bottos et al., 2018), and under low dispersal rates the importance of ecological drift can be increased (Vellend, 2010). Compared to bacterial community assembly, archaeal community assembly was driven primarily by stochastic processes across all soil horizons (Figure 7B). The population density and diversity of archaeal communities tend to be lower in soils than bacterial communities that could have increased the relative influence of drift on archaeal communities, as Vellend et al. (2014) suggested that ecological communities with smaller population size and/or lower diversity are more prone to drift.

In summary, the taxonomic and functional diversity of microbiomes varied markedly between different horizons of permafrost-underlain tundra soil. The composition and diversity of bacterial and archaeal communities were associated with vertical gradient in soil physico-chemical parameters; however, a large fraction of variation remained unexplained by these variables. The metagenomic approach employed in this study demonstrates that the distribution of genes involved in cycling of organic carbon and nitrogen was strongly influenced by horizons of permafrost soil. Our results also suggest that accurately predicting the changes in taxonomy and functions of permafrost soil microbiomes based on environmental characteristics may be limited, because compositional changes in permafrost soil microbiome are largely governed by stochastic processes. Together, these results provide important ecological insights about permafrost soil microbiome and their drivers, which will be helpful in understanding their responses to a warming climate.

## Data Availability

The datasets generated for this study can be found in NCBI Short Read Archive (SRA) and MG-RAST, SRA BioProject ID is PRJNA 513409 and MG-RAST project ID is mgp21063.

## Author Contributions

BT, HK, MK, and YL designed the research. HK, HJ, and SN completed fieldwork in Council, Alaska. JJ, SN, and YL contributed with reagents and analytical tools. BT, HK, and MK performed the research and analyzed the data. BT, MK, and YL wrote the first draft of the manuscript and all authors contributed to and have approved the final manuscript.

### Conflict of Interest Statement

The authors declare that the research was conducted in the absence of any commercial or financial relationships that could be construed as a potential conflict of interest.

## Footnotes

^1^https://www.mothur.org/wiki/Silva_reference_files

^2^https://www.mg-rast.org/linkin.cgi?project=mgp21063
