# Supplementary material for: Distinct Taxonomic and Functional Profiles of the Microbiome Associated With Different Soil Horizons of a Moist Tussock Tundra in Alaska
Source: Front Microbiol. 2019 Jun 27;10:1442. doi: 10.3389/fmicb.2019.01442 (PMC6610311; doi:10.3389/fmicb.2019.01442)
Supplement: Supplementary file 1 [file Data_Sheet_1.docx]

**Supplementary information**

**Distinct taxonomic and functional profiles of microbiome associated with different soil horizons of a moist tussock tundra in Alaska**

Binu M. Tripathi^a,1^, Hye Min Kim^b,1^, Ji Young Jung^a^, Sungjin Nam^a^, Hyeon Tae Ju^a^, Mincheol Kim^a,^*, Yoo Kyung Lee^a,^**

^a^Korea Polar Research Institute, Incheon 21990, Republic of Korea

^b^Environmental Safety Research Institute, NeoEnBiz, Bucheon 14523, Republic of Korea

*Corresponding author. Korea Polar Research Institute, Incheon 21990, Republic of Korea.

**Corresponding author. Korea Polar Research Institute, Incheon 21990, Republic of Korea.

*E-mail addresses:* [mincheol@kopri.re.kr](mailto:mincheol@kopri.re.kr) (M. Kim), [yklee@kopri.re.kr](mailto:yklee@kopri.re.kr) (Y.K. Lee).

^1^These authors contributed equally to this work.


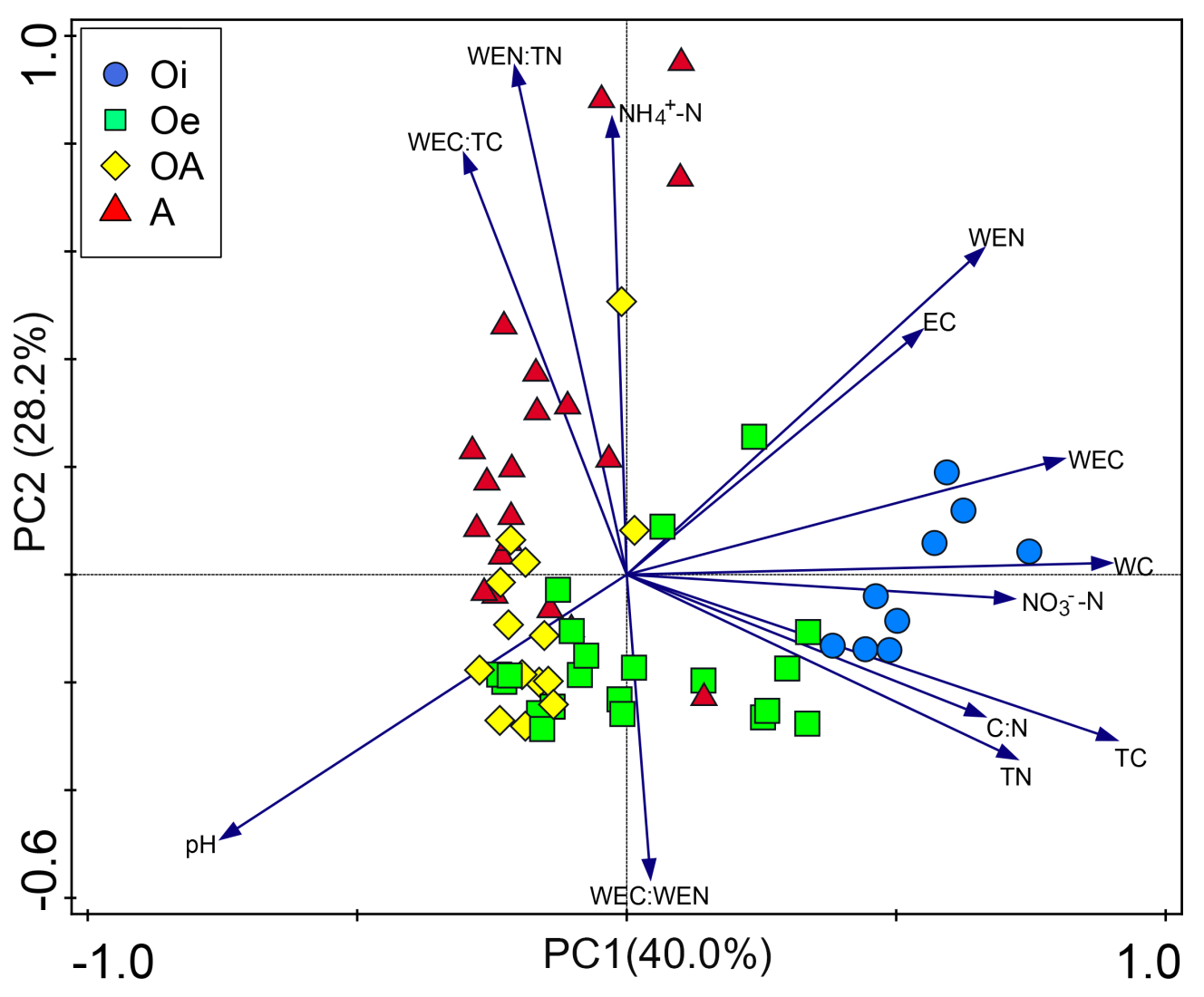


**Fig. S1.** Principal component analysis ordination plot on soil physico-chemical parameters.


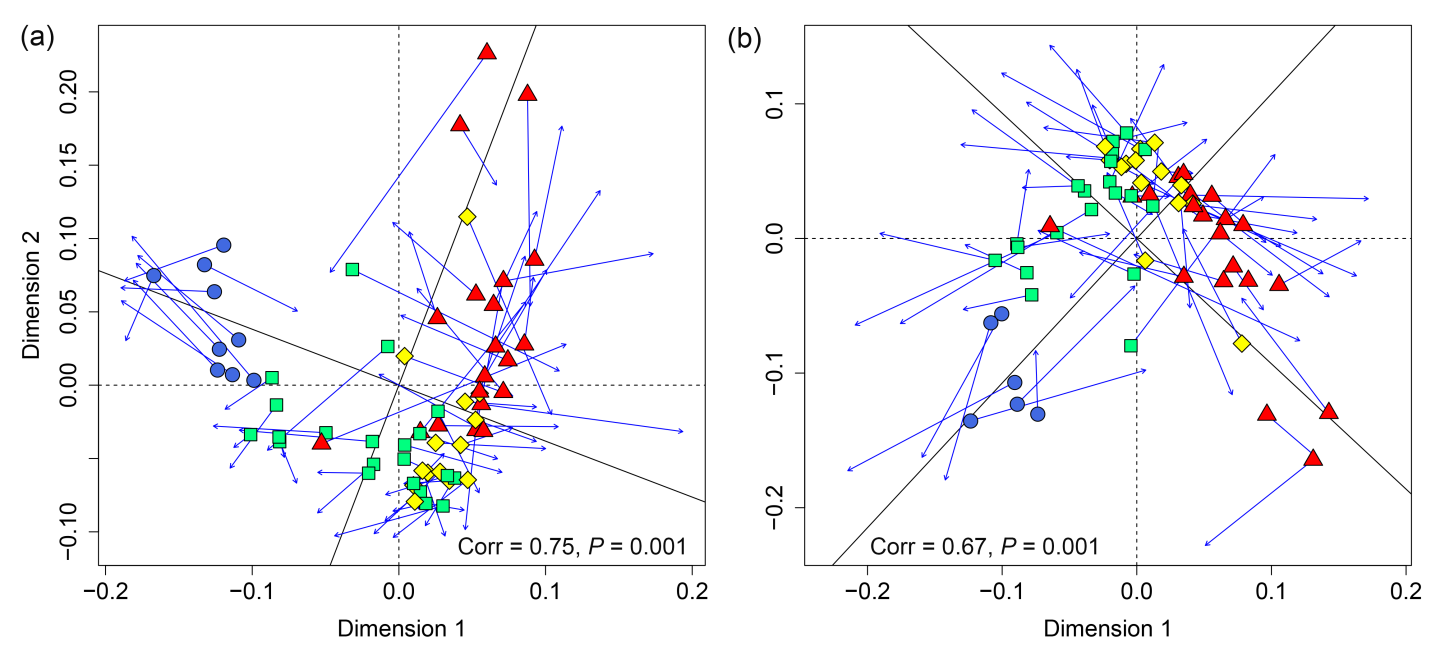


**Fig. S2.** Procrustes analysis comparing ordinations of (a) bacterial and (b) archaeal community structure and soil physico-chemical parameters. The arrows in Procrustes analysis point towards the target configuration (microbial community structure in NMDS plots), and symbols represent the rotated configuration (soil physico-chemical parameters in PCA plots).


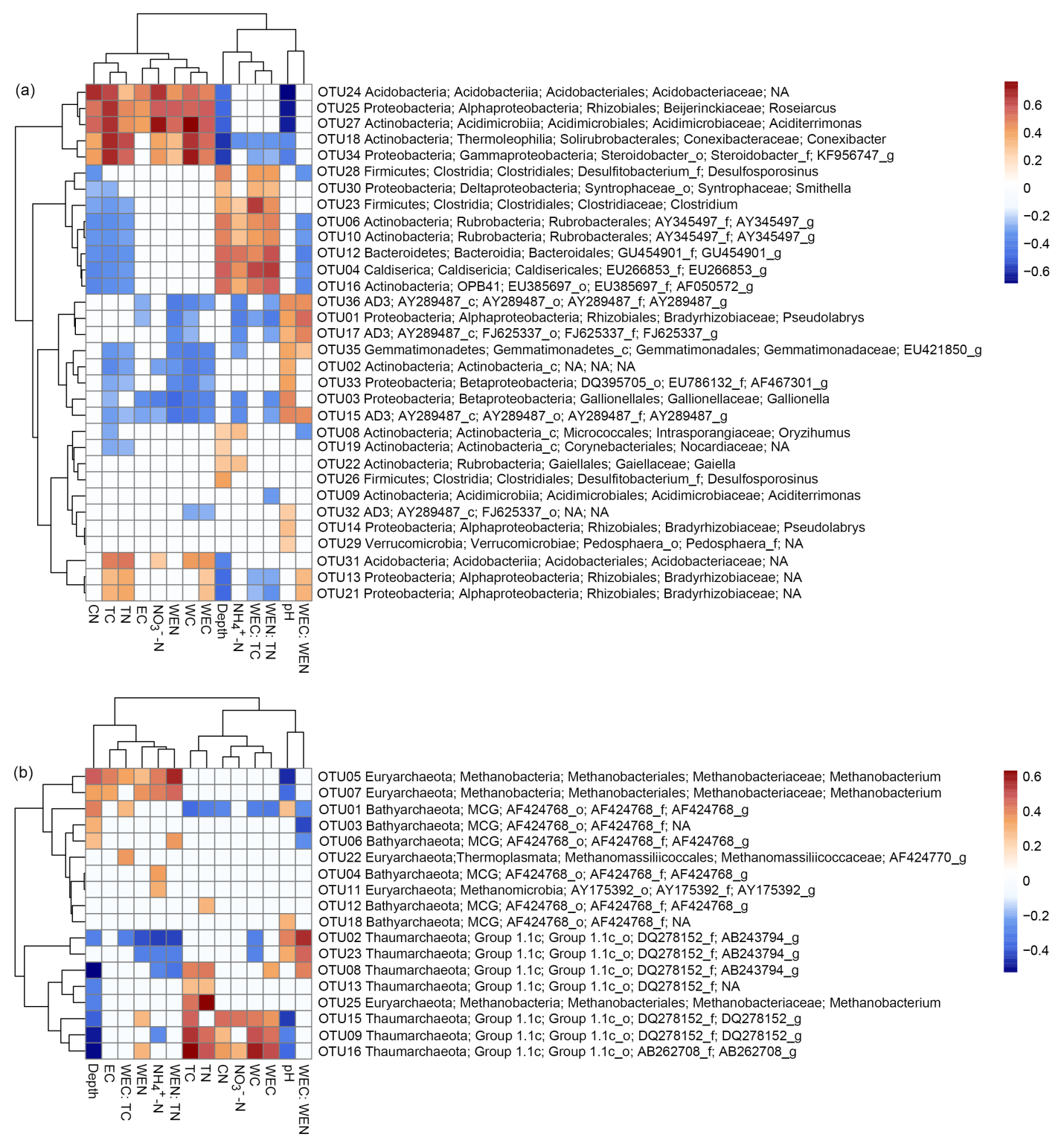


**Fig. S3.** Correlations between dominant (a) bacterial and (b) archaeal OTUs and environmental variables. Colors indicate the positive and negative Spearman’s correlation coefficients, as shown in color keys. “NA” in OTUs taxonomy indicates unclear classification. TC, total carbon; TN, total nitrogen; EC, electrical conductivity; WEN, water extactable nitrogen; WC, water content; WEC, water extactable carbon; pH, soil pH.


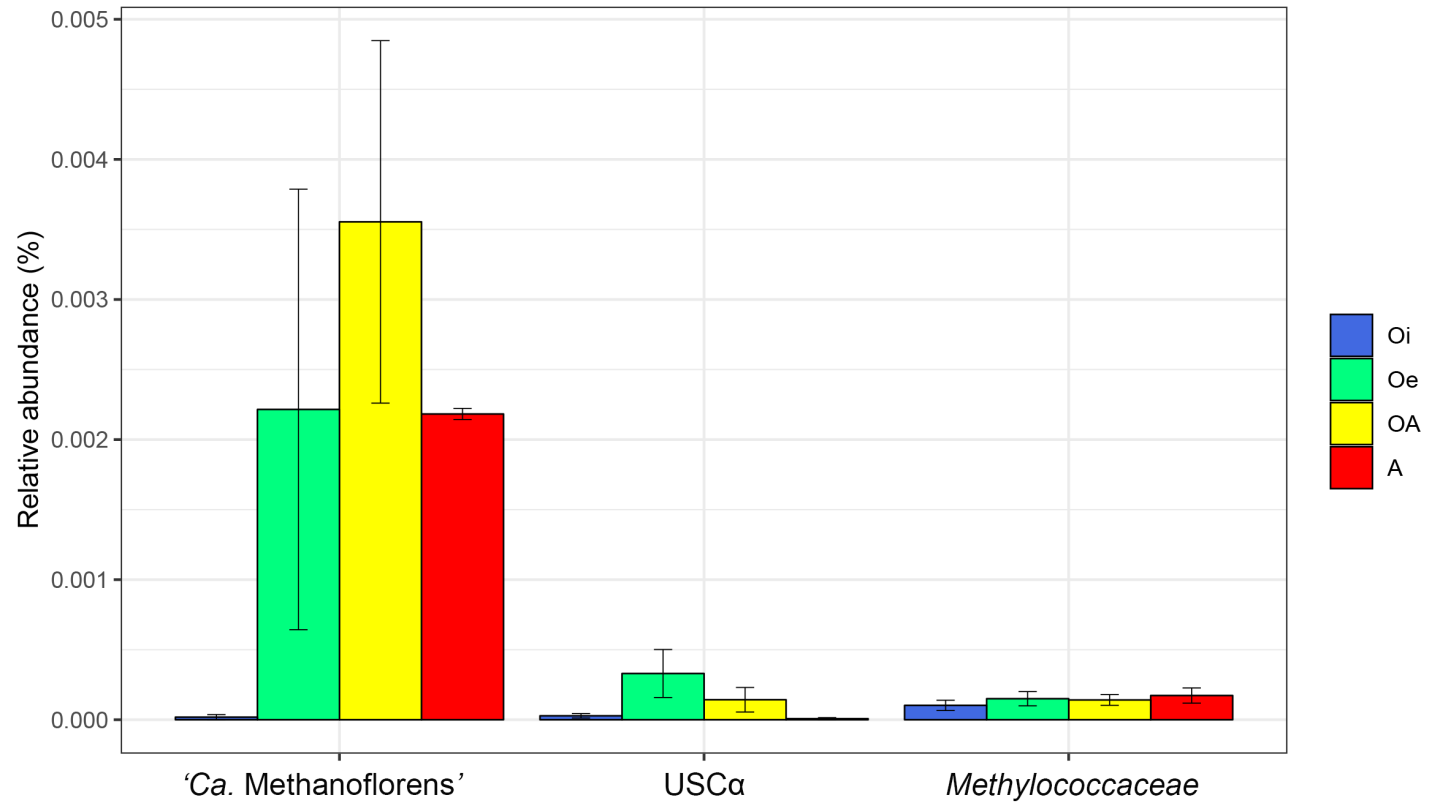


**Fig. S4.** Relative abundance of dominant methanogenic and methanotrophic taxa identified using *mcrA* (‘*Ca.* Methanoflorens’), *pmoA* (USCα) and *mmoX* (*Methylococcaceae*) specific GraftM packages.

**Table S1.** Soil physico-chemical properties of different soil horizons across all soil cores.

|  | Oi | Oe | OA | A |
| --- | --- | --- | --- | --- |
| Soil physico-chemical properties |  |  |  |  |
| pH | 4.08±0.25 (b) | 5.17±0.30 (a) | 5.15±0.29 (a) | 4.89±0.54 (a) |
| EC (µS/cm) | 551.00±499.71 (a) | 128.71±57.15 (b) | 129.71±101.87 (b) | 225.30±255.13 (b) |
| WC (%) | 444.56±172.45 (a) | 192.29±120.37 (b) | 82.00±47.94 (c) | 126.80±52.75 (b,c) |
| TC (%) | 41.43±2.91 (a) | 20.10±15.38 (b) | 5.72±3.01 (c) | 4.70±7.69 (c) |
| TN (%) | 0.99±0.22 (a) | 0.85±0.70 (a) | 0.23±0.13 (b) | 0.22±0.35 (b) |
| C:N | 43.48±9.45 (a) | 25.15±4.62 (b) | 24.91±4.02 (b,c) | 20.12±4.27 (c) |
| NO_3_^−^-N (μg N g^−1^ soil) | 1.07±0.57 (a) | 0.34±0.17 (b) | 0.22±0.15 (b) | 0.27±0.15 (b) |
| NH_4_^+^-N (μg N g^−1^ soil) | 15.02±13.09 (b) | 17.13±27.75 (b) | 21.99±27.34 (a,b) | 45.39±32.60 (a) |
| WEC (mg C g^−1^ soil) | 0.92±0.27 (a) | 0.53±0.31 (b) | 0.30±0.25 (b) | 0.34±0.21 (b) |
| WEN (mg N g^−1^ soil) | 0.04±0.02 (a) | 0.02±0.01 (b) | 0.01±0.01 (b) | 0.02±0.02 (b) |
| WEC:WEN | 25.06±6.06 (a,b) | 33.66±10.92 (a) | 28.04±12.91 (a) | 16.63±8.29 (b) |
| WEC:TC | 0.23±0.07 (b) | 0.36±0.27 (a) | 0.60±0.63 (a) | 1.31±0.98 (a) |
| WEN:TN | 0.41±0.17 (b) | 0.29±0.21 (b) | 0.54±0.40 (b) | 1.63±1.01 (a) |

Data represent mean ± standard deviation. Different letters (in brackets) within each row denote significant differences (*P* < 0.05) between groups based on one-way ANOVA and Tukey’s HSD test.

**Table S2.** PERMANOVA results showing effect of soil horizon on soil physico-chemical properties and microbial communities.

| Analysis | Metric | Soil horizon | | | | | | | | Coring point | |
| --- | --- | --- | --- | --- | --- | --- | --- | --- | --- | --- | --- |
|  |  | Main test | | Pair-wise test | | | | | | Main test | |
|  |  | Pseudo**-***F* | *P* (perm) | Oi - Oe | Oi - OA | Oi - A | Oe - OA | Oe - A | OA - A | Pseudo**-***F* | *P* (perm) |
| Soil physico-chemical properties | Euclidian | 16.1 | 0.0001 | 0.0001 | 0.0001 | 0.0001 | 0.002 | 0.0001 | 0.001 | 1.3 | 0.26 |
| Bacteria | Bray-Curtis | 8.0 | 0.0001 | 0.0001 | 0.0001 | 0.0001 | 0.001 | 0.0001 | 0.0001 | 1.1 | 0.26 |
| Archaea | Bray-Curtis | 4.7 | 0.0001 | 0.0004 | 0.0001 | 0.0002 | 0.102 | 0.0001 | 0.001 | 1.5 | 0.07 |

**File Name: Supplementary Data 1**

**Description:** The results of DESeq2 analysis across soil horizons for functional genes (KEGG level 3). *P*-values for multiple testing were corrected using the BH method. The function genes with *P*-values < 0.05 are only shown here.
